# From theory to practice: the inclusion of hospitalized children’s families in painful procedures

**DOI:** 10.1590/1980-220X-REEUSP-2023-0152en

**Published:** 2023-08-25

**Authors:** Danton Matheus de Souza, Rafaela de Fátima Fernandes, Cibelle Tiphane de Sousa Costa, Camila Amaral Borghi, Lisabelle Mariano Rossato

**Affiliations:** 1 Universidade de São Paulo, Escola de Enfermagem, Programa de Pós-Graduação em Enfermagem, São Paulo, SP, Brazil.; 2 Universidade de São Paulo, Escola de Enfermagem. São Paulo, SP, Brazil.; 3 Universidade Municipal de São Caetano do Sul, São Paulo, SP, Brazil.; 4 Universidade de São Paulo, Escola de Enfermagem, Departamento de Enfermagem Materno-Infantil e Psiquiátrica, São Paulo, SP, Brazil.

**Keywords:** Pain, Family, Hospitalized Child, Pain Management, Nursing, Qualitative Research, Dolor, Familia, Niño Hospitalizado, Manejo del Dolor, Enfermería, Investigación Cualitativa, Dor, Família, Criança Hospitalizada, Manejo da Dor, Enfermagem, Pesquisa Qualitativa

## Abstract

**Objective:**

To understand nursing team professionals’ strategies to include the family in painful procedures performed on hospitalized children.

**Method:**

An exploratory-descriptive, qualitative study, carried out with nursing professionals. Data were collected through semi-structured interviews, guided by a script of topics, transcribed and submitted to thematic content analysis, in the light of Symbolic Interactionism, discussed considering the Family-Centered Care philosophy assumptions.

**Results:**

Two central categories emerged, “Theoretical perspective: the family as a care agent in painful procedures” and “Practical perspective: experiences, challenges and strategies in painful procedures for family inclusion”, with their respective subcategories.

**Conclusion:**

Nursing professionals have theoretical knowledge about family inclusion in painful procedures based on the assumptions: Family-Centered Care: dignity and respect; information sharing; joint participation; and family collaboration. However, knowledge is not applied in clinical practice; consequence of the interaction between beliefs and attitudes unfavorable to family presence.

## RESUMO

**Objetivo:**

Compreender as estratégias dos profissionais da equipe de enfermagem para a inclusão da família nos procedimentos dolorosos realizados em crianças hospitalizadas.

**Método:**

Estudo exploratório-descritivo, qualitativo, realizado com profissionais de enfermagem. Os dados foram coletados por meio de entrevistas semiestruturadas, guiadas por um roteiro de tópicos, transcritas e submetidas à análise temática de conteúdo, à luz do Interacionismo Simbólico, discutidas frente aos pressupostos da filosofia do Cuidado Centrado na Família.

**Resultados:**

Emergiram-se duas categorias centrais, “Perspectiva teórica: a família como agente de cuidado nos procedimentos dolorosos” e “Perspectiva prática: vivências, desafios e estratégias nos procedimentos dolorosos para inserção da família”, com suas respectivas subcategorias.

**Conclusão:**

Os profissionais de enfermagem possuem conhecimento teórico sobre a inclusão da família no procedimento doloroso com base nos pressupostos: Cuidado Centrado na Família: dignidade e respeito; compartilhamento de informações; participação conjunta; e colaboração da família. No entanto, os conhecimentos não são aplicados na prática clínica; consequência da interação entre as crenças e atitudes desfavoráveis à presença da família.

## RESUMEN

**Objetivo:**

Comprender las estrategias de los profesionales del equipo de enfermería para incluir a la familia en los procedimientos dolorosos realizados en niños hospitalizados.

**Método:**

Estudio exploratorio-descriptivo, cualitativo, realizado con profesionales de enfermería. Los datos fueron recolectados a través de entrevistas semiestructuradas, guiadas por un guión de temas, transcritas y sometidas al análisis de contenido temático, a la luz del Interaccionismo Simbólico, discutido frente a los presupuestos de la filosofía del Cuidado Centrado en la Familia.

**Resultados:**

Surgieron dos categorías centrales, “Perspectiva teórica: la familia como agente de cuidado en los procedimientos dolorosos” y “Perspectiva práctica: experiencias, desafíos y estrategias en los procedimientos dolorosos para la inclusión familiar”, con sus respectivas subcategorías.

**Conclusión:**

Los profesionales de enfermería poseen conocimientos teóricos sobre la inclusión de la familia en el procedimiento doloroso a partir de los supuestos: Cuidado Centrado en la Familia: dignidad y respeto; el intercambio de información; participación conjunta; y colaboración familiar. Sin embargo, el conocimiento no se aplica en la práctica clínica; consecuencia de la interacción entre creencias y actitudes desfavorables a la presencia de la familia.

## INTRODUCTION

Child hospitalization generates impacts on families. Parents, in particular, experience an emotional turmoil, facing the transfer of their child’s care to health professionals, with renunciation of the parental role of caregiver to the acquisition of a passive role, especially if professionals are not favorable to their inclusion^(1,2)^. Aiming to change this context, health institutions have included the philosophy of Family-Centered Care (FCC) in their practices. This philosophy aims at planning, delivering and assessing health care based on mutually beneficial partnerships between health professionals and patients, of all ages, and families, at all levels of care^(3)^.

FCC emerged in the mid-50s, due to the need to rethink care for hospitalized children. At that time, children were hospitalized in the absence of their family, who could visit on certain days and times, generating the phenomenon known as hospitalism, with biopsychosocial and spiritual damage resulting from this breach of bond^(4)^. From the 50s onwards, initiatives emerged to change this context, moving from flexible visits to the right to stay with children^(4)^. In 1992, the Institute for Family Centered Care was created, a pioneering organization in the establishment of FCC and initiatives for its dissemination^(3,4)^, making its assumptions (dignity and respect; sharing information; family joint participation; and family collaboration) into core competencies of pediatric nurses^(3)^.

Despite the advances, in clinical practice, there are impasses in FCC implementation, especially in painful procedures, which are experienced in the hospital routine as part of diagnosis and treatment. In a study^(5)^ carried out in a Neonatal Intensive Care Unit with 90 newborns followed for three days, exposure to 2732 painful procedures was observed, with an average of 30 procedures per newborn^(5)^. Often, the family is excluded from these procedures, but the results of an investigation revealed that 94% of parents wish to remain in this context^(6)^.

Considering this issue, international bodies have dedicated themselves to reformulating ideal pain management (assessment, intervention and reassessment), mainly in procedures. In a family concept analysis of pain management, genuine member participation, with collaborative communication, individualized in a safe environment and with the dyad inclusion (children, family and professional) at the center of the process, was portrayed as an essential attribute for the management stages to occur successfully^(7)^. This participation promotes a redefinition of the family from passive to active in care, which is consistent with the FCC principles^(3,7)^.

Over the past twenty years, research and international bodies have demonstrated the benefits of including the family in invasive procedures and have taken a position in favor^(1–3,6)^. However, this advance did not guarantee a translation of knowledge to our clinical practice, noting a frequent family exclusion. Thus, the following questions emerged: what are the strategies carried out by professionals from the nursing team to include family members of children in the management of procedural pain during hospitalization? How does this inclusion occur in clinical practice?

Knowing this context allows identifying the main gaps in care practices and reformulating strategies, aiming to ensure family presence in painful procedures and alleviate the pain of children in this context, with a mutually beneficial partnership. Thus, this article aimed to understand nursing team professionals’ strategies to include the family in painful procedures performed on hospitalized children.

## METHOD

### Study Design

This is an exploratory-descriptive study, with a qualitative approach^(8)^.

### Research Site and Population

The study was carried out at the Pediatric Inpatient Unit (PIU) of a secondary teaching hospital in the city of São Paulo. This institution’s philosophy of care is based on the FCC assumptions, with an incentive to value the family’s participation in care to ease the stress and suffering arising from the experience of illness and hospitalization.

### Eligibility Criteria

The research participants were professionals from the nursing team (nurses and nursing technicians) from different shifts who work in the care of children hospitalized for more than five years. Exclusion criteria were not established.

### Data Collection

Data were collected in August 2021. Nurses and nursing technicians were selected through convenience sampling, invited to participate in the survey in person, with joint reading, and signature of two copies of the Informed Consent Form (ICF). After acceptance, a sociodemographic characterization form was filled out (age; sex; title; and length of professional experience in hospital services with assistance to children). Afterwards, a semi-structured interview was started, guided by a script of topics, with the following open questions: how do you perform a painful procedure on children who are accompanied by the family? Could you explain to me what your actions are when performing a painful procedure on children who are accompanied by the family? What do you do to make the family member more participatory during a painful procedure?

The interviews were conducted by a male researcher, a nurse in the process of specialization in children’s and adolescents’ health, who had previous experience in qualitative research and conducting interviews. He was immersed in the nursing team at the time of research, but this did not influence the conduct of interviews and data analysis. The interviews were carried out in a private place, guaranteeing anonymity.

Thus, 15 professionals were interviewed; there was no refusal to participate; and there were no repeated interviews. The audios were recorded using two devices (a cell phone and an audio recorder), in order to guarantee the fidelity of speeches for later transcription. A total of 278 minutes were recorded, with time ranging from 10 minutes and 43 seconds to 34 minutes and 24 seconds. All interviews were transcribed by a pair of researchers for later analysis, not being sent to participants.

The theoretical data saturation technique^(9)^ was used, with the completion of data collection at the time the study’s objective was answered, with no addition of questions and themes that could contribute to phenomenon exploration. This saturation was previously discussed between two researchers. It should be noted that what Minayo^(10)^ brings in her work was used as a basis: “*An ideal qualitative sample is one that reflects, in quantity and intensity, the multiple dimensions of a given phenomenon and seeks the quality of actions and interactions throughout the process*”.

### Methodological Framework

Data were analyzed in the light of the Symbolic Interactionism (SI) theoretical framework^(11)^, which seeks to interpret the meanings that the studied phenomenon provides in the investigated subject, approaching, in addition to the lexical discourse, their interactions with the other and with their context. Subjects’ interaction with the phenomenon allows the formulation of perceptions, meanings and beliefs that directly influence their speech and should be valued. In the present study, the subjects (nursing team) interact directly with the phenomenon (family and painful procedure), influenced by their interaction with themselves (self) and with their context of action (PIU), thus producing symbols and interactions (perceptions, strategies and actions).

Furthermore, for treatment and discussion of the findings, we relied on the Institute for Family-Centered Care’s FCC theoretical approach^(3)^, emphasizing its assumptions: dignity and respect for the family (beliefs, values, culture, decisions and presence, ensuring dyad dignity in care); sharing information with the family (impartial communication); family joint participation (inclusion in decision-making); and family collaboration (inclusion in care, with a partnership with professionals).

### Data Analysis

Data were analyzed using Bardin’s thematic content analysis^(10)^. The material was transcribed and analyzed, in search of understanding, by cores of meaning, from codifications. Such codes were grouped, and became categories and subcategories, improved and grouped in theoretical categories. They were interpreted based on the study’s objective, theoretical framework and theoretical approach. In the end, two main theoretical categories and five interconnected subcategories emerged.

### Ethical Aspects

This is a subproject linked to the investigation “*Experiência da família e da equipe multiprofissional de saúde acerca do manejo da dor da criança hospitalizada*”, approved by the Research Ethics Committee of the Nursing School, *Universidade de São Paulo*, under Opinion 2.157.167, and by the co-participating institution, under Opinion 2.181.403, both in July 2017. The ethical principles of Resolution 466/12 of the Brazilian National Health Council were respected. Mothers’ discourses are identified throughout the text with the letter “I” (interviewee), followed by the number according to the entry into the study: I1, I2, I3...

## RESULTS

### Participant Characterization

In total, 15 nursing professionals participated in the study, five nurses and 10 nursing technicians. Participants’ age ranged between 30 and 45 years, with an average of 40 years, with 14 female participants. Among the nursing professionals, four had *stricto sensu* graduate degrees, and all had *lato sensu* degrees, with more than five years of experience in the sector. The nursing technicians had a long length of hospital service, ranging from 10 to 19 years of activities.

From the analysis of interviews, two central categories emerged, “Theoretical perspective: the family as a care agent in painful procedures” and “Practical perspective: experiences, challenges and strategies in painful procedures for family insertion”, with their respective subcategories.

#### 1) Theoretical Perspective: the Family as a Care Agent in Painful Procedures

**Chart 1:**
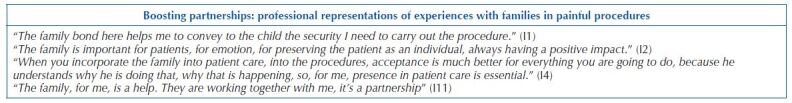
Discourses referring to the subcategory “Boosting partnerships: professional representations of experiences with families in painful procedures”. São Paulo, SP, Brazil, 2021.

In the subcategory “Boosting partnerships: professional representations of experiences with families in painful procedures” (Chart 1), it was observed that professionals recognize FCC as part of the organizational culture that impacts on assistance to the dyad, guiding their organization in painful procedures. They list, in their speeches, that family presence reduces emotional reactions, such as anxiety, for understanding the procedure, enhancing the bond with the team, with greater security, and with children, and being present even in times of difficulty. Children experience a sense of security, comfort, welcome and reassurance because their caregiver is supporting them, reducing trauma and the memory of pain, with the analogy of a mirror of reactions and impacts. Professionals feel safer at that moment, having established a partnership that will reduce the impact of painful procedures. Thus, there is a line of care that aims to establish a partnership, with positive representations of family’s presence, which impacts all agents present in a painful procedure.

**Figure 1:**
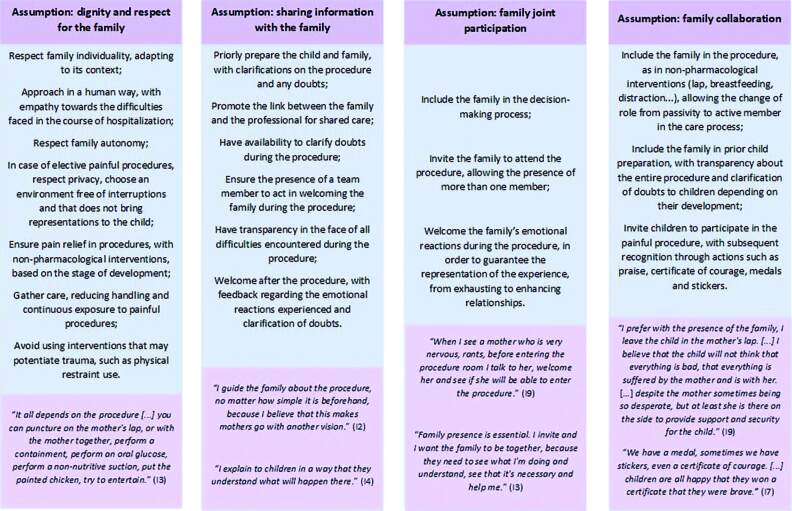
Strategies used by nursing team professionals to include the family in painful procedures based on the Family-Centered Care assumptions. São Paulo, SP, Brazil, 2021.

When asked about actions taken by FCC in painful procedures, professionals listed numerous strategies that are intertwined with the Institute for Family-Centered Care theoretical model assumptions (Figure 1). Professionals aim to guarantee family acceptance, with prior guidance, during and after, with active listening, clarification of doubts, sharing of decisions and respect for individualities, family presence throughout the process, encouraging them to actively participate in the procedure, and pain relief, with the very presence of a child safety figure and non-pharmacological intervention use. They interpret that all the time spent on the aforementioned strategies is an investment to provide dignified care (subcategory “Establishing a new care: strategies used by professionals to include the family in painful procedures”).

#### 2) Practical Perspective: Experiences, Challenges and Strategies in Painful Procedures for Family Insertion

Although professionals demonstrate theoretical knowledge, report being favorable to family presence in painful procedures and recognizing FCC as part of the institutional culture, when exploring their practical experiences, there are divergences in the translation of knowledge.

**Chart 2:**
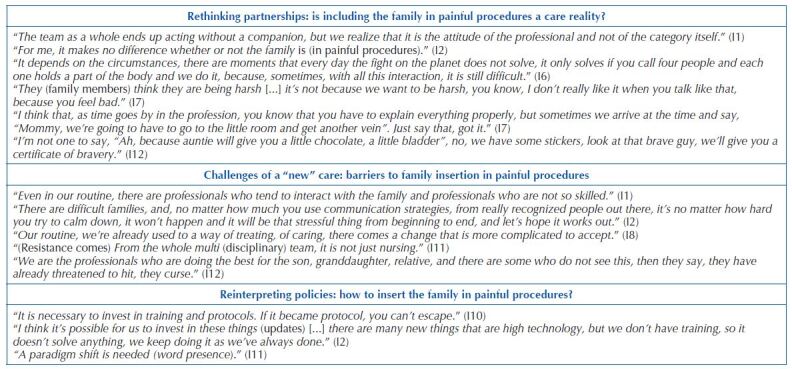
Reports referring to the subcategories of the category “Practical perspective: experiences, challenges and strategies in painful procedures for family insertion”. São Paulo, SP, Brazil, 2021.

In the subcategory “Rethinking partnerships: is including the family in painful procedures a care reality?” (Chart 2), professionals portray that including the family in procedures is an individual characteristic: if professionals are in favor of its inclusion, it will be present, if not, it will be excluded. Four participants reported that they are not in favor of family presence and that they feel discomfort, impotence, moral distress and discomfort. They report charges for the social representation of nursing team members as angelic and caring figures who need to be with the family all the time, aspect that causes wear and tear and accentuates indifference regarding their presence at the time of a procedure: the family member can accompany them, but no actions will be taken regarding their presence.

They portray that the family is represented by FCC as an always potential figure, but that, in clinical practice, there are numerous profiles of family members and that, for the most part, they are aggressive, hostile and hinder professionals in their work. In practice, when families show emotional reactions to the procedure, they are asked to leave, as they become a “burden” and one more agent to be cared for.

Painful procedures bring with it demands from the professionals themselves, mainly due to previous experiences in which the family represented them as a potentiating agent of trauma to children, generating charges, anxiety, experiencing threats, mistakes, stress and moral suffering, which contributes to not allowing partnership, listed in the theory, to occur in its entirety. They report that they only allow the presence of a family member, due to aggression experiences, when there is more than one member, and that information and guidance will only be provided if the procedure is elective, and, in case of emergencies, these are postponed. All of these listed aspects are conducive to conflicts and difficult relationships.

Professionals define FCC as a “new” policy, despite the fact that the right to a companion has been guaranteed for more than two decades and that there are professional, family and institutional barriers to translating the listed strategies. As for professionals, they refer: lack of skills and previous training; forgetfulness; mechanical assistance in the face of a routine to be followed, an aspect more present in the speeches of professionals with more time of experience; humor; overload; discouragement; professional specialty, in which nurses and their technical team are the ones who still carry out actions for inclusion; time spent on actions; collective and non-individualized planning; family stereotyping and belief in the existence of a hierarchy between its members and professionals; and resistance in the implementation of new strategies to improve care (“*I’ve always done it this way*”).

They list as family barriers: emotional reactions; accentuated stress; blaming and social representation of professionals; conduct without the family’s consent; professional approach with violent communication, and family conflicts. Institutional barriers report: absence of encouragement from superiors and permanent education; and unit dynamics that influences the planning of procedures and time spent (subcategory “Challenges of a “new” care: barriers to family insertion in painful procedures”) (Chart 2).

About professionals being asked about what could be done to ensure the translation of theoretical knowledge into clinical practice, they list: continuing education; formulation of protocols that guarantee the mandatory family presence; family meetings to know families’ individualities and convey them to care planning, and that they are interdisciplinary actions. Professionals mention that the policies that guarantee family presence as a companion and/or visitor should be emphasized, aiming at a reinterpretation of the word “presence”, since, in addition to the presence, Strategies, such as those mentioned in Figure 1, need to be implemented so that this experience is free of trauma and that, in fact, the family becomes a partner in child care (subcategory “Reinterpreting policies: how to insert the family in painful procedures?”) (Chart 2).

## DISCUSSION

It was observed that the interaction between nursing professionals, painful procedures in hospitalized children and families leads to two symbolic lines: in one, there is theoretical knowledge consistent with FCC, and in another, there are beliefs and attitudes unfavorable to the philosophy of care, permeating an ineffective application of knowledge.

The benefits of including the family in painful procedures are already reported in the literature^(3,12,13,14)^, corroborating this study. In a systematic review that aimed to assess families’ experiences in this context, it was observed that parents want to be present, because they believe in the benefits to the dyad, with emotional and physical security for children, in addition to reducing anxiety and increasing satisfaction with family care, and partnership with professionals^(6)^. Moreover, the presence can be considered a non-pharmacological intervention for pain relief in procedures. In a randomized clinical trial that assessed the effects of family presence, in comparison to child distraction (without parents), and of a group without any intervention, during venipunctures, it was observed that the presence was the most effective intervention, reducing vital signs and pain score (p < 0.001)^(2)^.

However, it is worth reflecting that, in addition to presence, it is necessary to expend strategies for the transition of the family’s role from passive to active in care, according to the FCC assumptions^(3)^. In this sample, professionals have knowledge consistent with care and recognize numerous strategies that they use to act. However, it is emphasized that the interviewee may have answered what the researcher wanted to hear; for this reason, we used SI as a theoretical framework, enabling a more critical analysis of the symbolic interactions intrinsic to the discourse, emerging practical reality, which does not match the reports of theoretical knowledge^(11)^.

We returned to reflection: is including the family in painful procedures a care reality? It is noted that FCC has become a catchphrase in participants’ reports, losing its real meaning. Professionals say that they carry out actions for family inclusion, but they are not clear that inclusion, in itself, is a family right, that FCC goes beyond that: it is not having mothers on children’s side, but a mutually beneficial partnership^(15)^. The same is seen in institutions, which define FCC as part of an institutional culture, however it is relatively easy to accept family participation and consider it as part of a philosophy that defends a broad collaboration change, starting from the assistance partnership to decision-making in institutional policies^(3)^. It is necessary to understand health professionals’ real perceptions to identify what are the impasses for the adequate FCC implementation.

The literature indicates that professional knowledge is a positive predictor of family inclusion in painful procedures^(16)^, which does not corroborate the results of this study. It is necessary to go further, because the construction of symbols is not only based on what individuals know, but also on what they experience and implicit interactions^(11)^. Other indicated predictors are working time and education^(13)^, but in this sample, there is a predominance of professionals with more than five years of experience and with a *lato sensu* graduate degree completed. To understand this scenario, it is necessary to address professional beliefs and attitudes.

Investigations report health professionals’ restrictive beliefs, such as the stereotype of a “difficult family”, questioning and demanding, who do not understand the procedure, anxious, that conveys this feeling to children, in addition to the suffering that the procedure will always cause the family, excluding them with the belief in protection and the fear of criticism of professional performance. In common, they result in the belief of the family disturbing the procedure, which, added to previous experiences, leads to exclusion as a protective measure^(2,6,12,17)^, corroborating the discourses of this study. This aspect is in accordance with a basic premise of SI: “*Human beings act according to the meaning they attribute to phenomena and the dispositions that individuals are in a given context, activity and institution*.”^(11)^.

In a historical review, it was observed in studies carried out in the 50s that nurses were not convinced that family inclusion was a good idea, with the belief that it would harm the relationship with professionals, being hostile to the idea^(4)^. However, despite the evolution, this study brings speeches from professionals who recognize themselves as not favorable to the family presence with attitudes of exclusion, even after so much scientific progress.

Another point for reflection on attitudes of exclusion is whether the nursing team recognizes them as a problem. In this study, respondents were not asked about their position towards the family, only about strategies and clinical practice, however, intrinsically, they listed these aspects. Starting from SI, human action is the result of the interaction between all situations experienced. There is an interaction between professionals, generating a society (nursing team) that is marked by a culture (family presence or exclusion)^(11)^. This aspect reiterates the need to reflect the context beyond the isolated action of professionals.

Professionals consider FCC a “new” philosophy, and the quote game brings this reflection. As highlighted in the introduction to this article, FCC emerged in the mid-50s and established itself in the 90s^(3,4)^, not being a new philosophy, but a way of caring that has not yet been established in health services in our context, reaffirming the need for this study. The Institute for Family Centered Care already considered this aspect in 2008, reiterating that, even if institutional policies occur, the hospital culture takes time to change^(3)^, however, in 2021, the family is still left in the background.

SI reiterates that human behavior is not only influenced by what happened in the past, but also by what happens and is experienced in the present^(11)^; in this case, professionals’ speeches may have been influenced by the experience of the COVID-19 pandemic, when families were excluded from adult care and limited in the presence of accompanying children^(18,19)^. This aspect may have influenced the view, with the hypothesis that professionals have readjusted to a reality in which family presence is not an option.

Professionals recognize personal, family and institutional barriers to family inclusion as well as results of interaction with the phenomenon, which should be added to all the findings presented in the reflection of change strategies^(20,21)^. In particular, a barrier to highlight is difficult relationships. In this study, professionals mention that FCC recognizes the family as an always potential figure, and this aspect must be highlighted. In clinical practice, there are numerous profiles, behaviors and emotional reactions, with various antecedents, on both sides of the story, which must be respected. A family conflict emerges from the sum of stressful factors, requiring recognition of the family’s individuality and the exposed determinants, in order to shape care based on individual needs^(3)^.

It is known that family partnership in painful procedures is not a reality in all contexts. Individuality returns as an essential point. There are families who are absent, who do not want to participate, and who, regardless of the approach, are aggressive and disrespectful. However, as long as FCC is likely to occur, priority should be given to establishing this partnership^(3)^. Still, in cases of absence, professionals can spend non-pharmacological interventions and recognition of children as an active member in the procedure^(22)^, as listed in Figure 1.

A mutually beneficial and culturally responsive partnership is the proposed goal for alleviating pain and trauma from painful procedures. Here, partnership is understood as a respectful, trusting collaboration, with agreed objectives, shared responsibilities and capabilities, recognizing that family members are essential to transformational changes in health care, leading to safety, quality and satisfaction^(3,23)^.

The American Academy of Pediatrics released recommendations for family presence in painful procedures and emergency care, namely: consider presence as an option; previously assess members’ conditions, in search of something that could affect their inclusion; pre-define who will assist in care; consider professionals’ safety; respect individualities; and document the procedure, citing the strategies used in care^(24)^. In this context, it is worth reflecting on measures to make it possible to translate this care into clinical practice.

The professionals in this study listed numerous possibilities to change the current context, according to the subcategory “Reinterpreting policies: how to insert the family in painful procedures?”. In addition to these, the literature indicates the following strategies: professionals’ active education, with simulations; inclusion of family participation as an indicator of quality; staff awareness programs; continuity of scientific research, with interdisciplinary implementation studies; and partnership at different levels of action, with involvement of the dyad, administrators, planners, policy makers and government agencies, with a long-term commitment^(3,12,15,25)^. The actions listed by professionals (Figure 1) are of immense value, but must be considered so that they are actually used in clinical practice.

In order to choose the best strategy, professionals must reflect on the context in which they operate, due to the interactions that influence the phenomenon’s symbolic interpretation and implementation constancy, as professionals can change their behavior for a period and then resume excluding practices. Nurses and their technical team can act in this line of care with the basics: being favorable to the family’s partnership in a painful procedure, because, in the end, FCC will be an individual characteristic. Consequently, the assumptions and strategies indicated will be consequences of a collective effort.

This study presented as limitations the absence of possibilities of generalization of the findings, the inclusion only of professionals from the nursing team, considering that the work with FCC must be an interdisciplinary practice, and the limitation to only one hospital context. However, it is expected that the study can guide reflections on the family’s partnership in painful procedures so that the inclusion strategies mentioned can be, in fact, applied in institutions and that future research will expend actions to change this context.

## CONCLUSION

It was observed in this study that nursing professionals have theoretical knowledge about family inclusion in a painful procedure, listing numerous strategies consistent with the FCC assumptions; however, in their practice, there is difficulty in the application of knowledge due to the interaction between beliefs and attitudes, generating symbols that are unfavorable to this philosophy of care.
